# Dogs functionally respond to and use emotional information from human expressions

**DOI:** 10.1017/ehs.2022.57

**Published:** 2022-12-06

**Authors:** Natalia Albuquerque, Briseida Resende

**Affiliations:** Institute of Psychology, University of São Paulo, São Paulo, Brazil

**Keywords:** *Canis familiaris*, dog–human relationship, emotion perception, emotion regulation, social cognition

## Abstract

Emotions are critical for humans, not only feeling and expressing them, but also reading the emotional expressions of others. For a long time, this ability was thought to be exclusive to people; however, there is now evidence that other animals also rely on emotion perception to guide their behaviour and to adjust their actions in such way as to guarantee success in their social groups. This is the case for domestic dogs, who have tremendously complex abilities to perceive the emotional expressions not only of their conspecifics but also of human beings. In this paper we discuss dogs’ capacities to read human emotions. More than perception, though, are dogs able to use this emotional information in a functional way? Does reading emotional expressions allow them to live functional social lives? Dogs can respond functionally to emotional expressions and can use the emotional information they obtain from others during problem-solving, that is, acquiring information from faces and body postures allows them to make decisions. Here, we tackle questions related to the abilities of responding to and using emotional information from human expressions in a functional way and discuss how far dogs can go when reading our emotions.

## Graphical abstract

**Figure:**
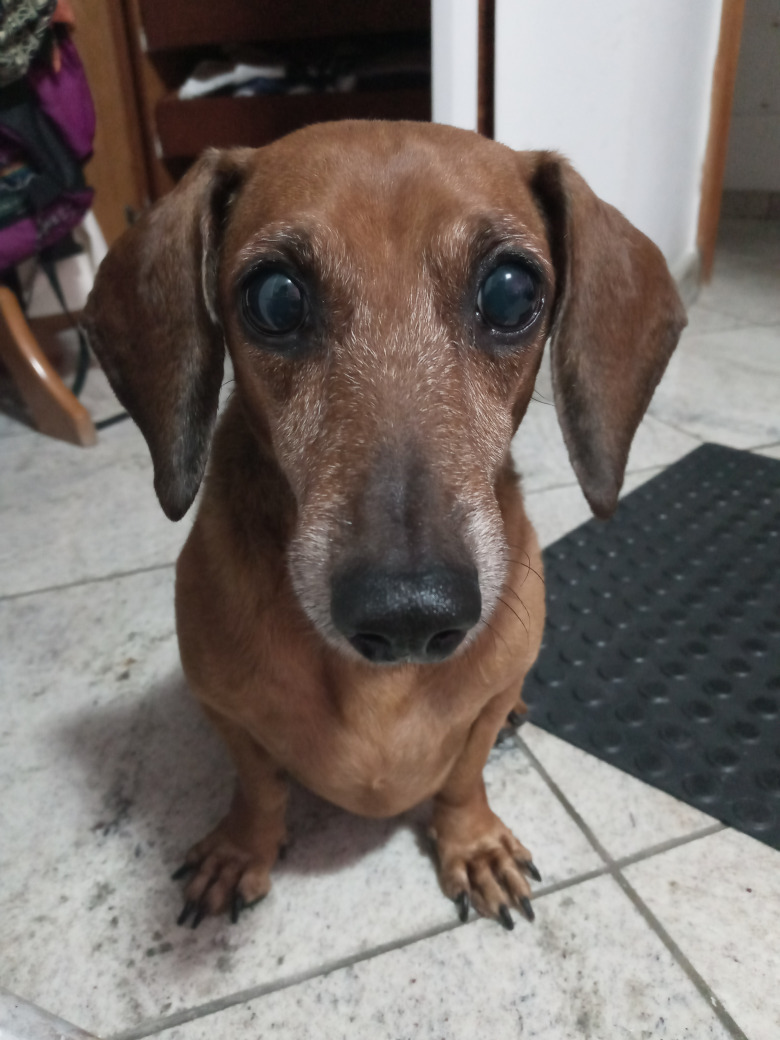

**Social media summary:** dogs recognise and infer emotional information from humans and use this information to regulate their own behaviour.

## 1. Introduction

The emergence and the development of the ability to read emotions from individuals bring clear evolutionary advantages. Conversely, the mechanisms behind the ability to read emotions from heterospecifics are less clear. When thinking of domestic dogs, however, being able to recognise the emotional expressions and states of a different species, for instance humans, might be vital for functional social interactions. Living in a complex social world requires sophisticated knowledge about other individuals and this information is what allows animals to predict the behaviour of others (Bugnyar & Henirich, 2005). Social information is critical to adult humans in various ways, including making decisions and defining whether an individual might gain help or resources from others or not (Milinsk, 2016), and the same might be true for dogs.

For Marc Bekoff (2000), emotions can be broadly defined as complex multifaceted psychological phenomena that influence the management and the control of behaviour. Emotions play such a crucial role that their experience, in terms of both quality and intensity, can interfere in how events are registered in our memory (Ades et al., 1990).

Dogs, in their day-to-day lives, are not only passive of their own emotional experience but are also active subjects for expressing their emotions in a communicative way and, further, for recognising the emotions and emotional expressions of others. Dogs are very well adjusted to their multispecies groups, families and life dynamics. However, to what extent does appropriately perceiving the emotions of the people they interact with play a critical role to their success? Does reading emotions allow them to live functional social lives? In this paper, we aim at tackling questions related to dogs’ emotion perception abilities from a functional approach. First, we will bring to light the dog–human relationship, then we will follow with a discussion of the functional perspective on emotion perception and finally we will show evidence that dogs respond to and use implicit information from human emotional expressions.

## 2. Dogs and humans: More than a bond

A great deal of dogs’ social living occurs in mixed-species groups (Miklósi, 2008). In fact, humans and dogs are more than sympatric species, i.e. there is more than territory overlap in place. In fact, they establish long lasting, dynamic, complex and mutually advantageous relationships (Albuquerque & Ciari, 2013). These two species have co-existed for at least 10,000 years (Pendleton et al., 2018) with genetic evidence suggesting more than 20,000 years of divergence between the ancestor of the modern grey wolf and the ancestor of the domestic dog (Skoglund et al., 2015). During this shared evolutionary history, dogs may have been selected, probably unintentionally, for handling the complexities of heterospecific social relationships, with evidence supporting the hypothesis that they have developed different mechanisms to facilitate interaction with people (e.g. Nagasawa et al., 2015). The interspecific relationship between dogs and humans seems to be unique within the animal kingdom, with no other domestic animal having shared more of their evolutionary history in close contact with humans (Pendleton et al., 2018), and its benefits are of great social, health and economic relevance (e.g. Mills & Hall, 2014; Savalli & Ades, 2015).

Domestic dogs are known to be very good readers of human communicative cues such as pointing and looking (e.g. Ford et al., 2019), even from very young ages (Hare et al., 2002) or with little experience with people (Riedel et al., 2008). Dogs have also been shown to be sensitive to people's attentional state, showing distinct behaviour depending on the person's attention direction and attention availability (e.g. Call et al., 2003; Bräuer et al., 2004; Kaminski et al., 2009; Savalli et al., 2013). In addition, dogs have been found to be sensitive to ostensive directional signals, showing better performance in social tasks when communicative cues are presented in combination with them (Téglas et al., 2012) and not understanding cues as communicative when they are not directed at the subjects (Kaminski et al., 2012). Furthermore, Savalli et al. (2014, 2016) discuss that dogs also produce communicative signals and they do so in a functionally referential and intentional way.

Even though most studies are done with family dogs, i.e. dogs that live with a human family in a household, stray dogs represent more than 80% of the global dog population (Cabral & Savalli, 2020). These animals, so-called free-ranging dogs, are not restricted by human activities and are not under direct human care. They are seen living on the streets as scavengers and are very common in developing countries (Majumder et al., 2014) such as India and Brazil. To date, a few studies have been conducted with these animals and have found evidence for their complex cognitive abilities. For instance, they are capable of assessing the quantity of opponents in intergroup conflicts (Bonanni et al., 2010), assessing the intention (friendly vs. threatening gestures) of humans in a food provisioning task (Bhattacharjee et al., 2018) and using information provided by human pointing distal cues (Bhattacharjee et al., 2020), among others.

Emotional cues, however, are more subtle and their perception can comprise different processes. Despite its recency, the study of emotion perception in animals, especially non-primates, has been growing strong and rapidly. For instance, studies have shown that dogs present cognitive biases when exploring faces and show differential visual processing when presented with human or dog faces (Racca et al., 2012; Somppi et al., 2016). Dogs have been empirically shown to be particularly sensitive to human emotions (Kujala, 2018; Albuquerque, 2017). They discriminate and show differential responses to emotional cues expressed through body postures, facial expressions, vocalisations and odours (Vás et al., 2005; Müller et al., 2015; Albuquerque et al., 2016; Caeiro et al., 2017; D'Aniello et al., 2017), and emotional cues can influence their behaviour (e.g. Merola et al., 2012a; Albuquerque et al., 2021; Bremhorst et al., 2021). Moreover, current research has shown that cultural as well as developmental factors can influence these abilities (Katayama et al., 2019; Amici et al., 2019; Bolló et al., 2020).

## 3. Functional perspective of emotion perception

A functional approach refers to the history of the behavioural trait as well as to the consequences of possessing such a trait (Keltner & Haidt, 1999). The use of emotional information from conspecifics and, in the case of the dog, heterospecifics, may be seen as an adaptation for handling the complexities of the social environment, regardless of its underlying causes. For instance, affiliative behaviours are more flexible when they involve strategic decision-making, i.e. making choices conditional to the behaviour of the members of one's group (Hall & Brosnan, 2016). Therefore, anticipating someone's future behaviour and being able to respond accordingly is cognitively demanding and highly advantageous.

There is enough evidence to allow conclusions about the expression and the perception of emotions by dogs. Several animal species are known to be sensitive to emotions (e.g. Proops et al., 2018; Nawroth et al., 2018; Albuquerque et al., 2016). However, being able to obtain information from emotional expressions is not necessarily functional. Dogs’ social cognition facilitates the interaction with humans, and the ability to read and respond appropriately to emotional cues may have been – and may still be – key for the establishment of these interspecific bonds. In this context, it becomes crucial to investigate how dogs respond to emotional expressions and whether and how dogs use the emotional information from others in social situations mediated by distinct emotional valences.

From an evolutionary perspective, expressing and perceiving emotions becomes adaptive when the receiver uses the emotional information conveyed by the signaller to solve problems and to guarantee their success over ecologically relevant resources, i.e. one must use the emotional information in a way that increases fitness (e.g. feeding, monopolising food patches, finding mating partners), otherwise expressing and perceiving emotions will not be positively selected. According to van Kleef (2009), emotional expressions may affect an observer by triggering inferential processes and/or affective reactions in them and can benefit the receivers of the information with inputs on their decision-making.

Social life allows individuals to benefit from using public information and learning through socially mediated processes (Kendal et al., 2009; Resende et al., 2021) in various activities, such as feeding, mating, tool-use and cooperating, and these abilities are spread over the animal kingdom. For instance, human infants assess individuals by their behaviour towards others (Hamlim et al., 2007) and can selectively evaluate social interactions (Hamlim et al., 2011); great apes and monkeys can distinguish cooperative from non-cooperative parties (Call et al., 2004; Phillips et al., 2009), allocate differential attention to individuals depending on their role in social contexts (McFarland et al., 2013) and use social information of group members to adjust foraging strategies (Loreto, 2015); and dogs go in a similar direction, having been shown to be capable of using publicly available information (discerning the intention of different people in food-sharing interactions) and discriminating helper individuals from non-helpers (Marshall-Pescini et al., 2011; Chijiwa et al., 2015). Furthermore, a few studies have looked at the predictive use of emotional cues, with data showing that different animal species can use affective cues to direct their own behaviour (Waller et al., 2016; Buttelmann et al., 2009; Morimoto & Fujita, 2012; Buttelmann & Tomasello, 2013).

The relationship between dogs and humans is extremely relevant for the study of the evolution of social cognition in cooperative contexts owing to all its particularities (e.g. Savalli & Albuquerque, 2017). According to Keltner and Haidt (1999), functional explanations at group levels focus on how social information impacts the interaction between individuals who share common goals. Moreover, the functional approach allows the investigation of the function of emotions regardless of the cognitive construction of emotional states (Farb et al., 2013).

The ability to obtain information from faces is considered to be one of the major reasons for humans thriving as social animals (Fantz, 1964). Facial recognition may be divided into different components, including the ability to recognise facial expressions and to access the information within them (Johnson & de Haan, 2015). According to Ferretti and Papaleo (2018), recognising emotions presumes the encoding of multimodal sensory information to provide cues about the emotional states of another individual. Taking from its complex attributions, recognising emotions from faces has long been associated with animals with a large repertoire of facial expressions, such as humans and non-human primates. However, owing to its great adaptive value, especially towards mediating affiliative behaviours, avoiding harmful interactions and, thus, increasing survival chances, this ability seems to be advantageous for many groups of animals (Ferretti & Papaleo, 2018).

In 2018, Albuquerque and colleagues investigated the functional response to emotional expressions. They used dogs’ well-known behaviour ‘mouth-licking’ (i.e. licking one's own mouth) as a model, testing whether dogs reliably respond to emotional expressions. They found that mouth-licking was exhibited significantly more towards human faces showing negative emotional expression compared with happy faces. These findings show functional responses to emotional information by dogs and suggest that dogs have a functional understanding of emotional expressions.

Moreover, a few studies have found that dogs show physiological changes when presented with emotional expressions. For example, Yong and Ruffman (2014) revealed that the cortisol levels of dogs increased after listening to crying human infants and Siniscalchi et al. (2018a) found that domestic dogs show asymmetric engagement of brain hemispheres linked to physiological changes, with the prevalent use of the right hemisphere when processing negative vocalisations and the left hemisphere when processing positive valence.

Heart rate, heart rate variability and other measures such as body temperature have been used to assess the physiological responses of dogs to pleasant and aversive stimuli (e.g. Travain et al., 2016; Riemer et al., 2016). These measures have also been used to investigate dogs’ reactions to more subtle affective stimuli such as facial expressions with different emotional content (e.g. anger, happiness, sadness), and have provided data on differential physiological reactions to emotionally charged stimuli (Siniscalchi et al., 2018b) and that these responses are significantly affected by the subjects’ ontogenetic experiences (Barber et al., 2017). Furthermore, oxytocin levels are an important physiological measure of emotional expression in dogs (Mitsui et al., 2011). They also modulate the way dogs perceive human faces and, therefore, human facial expressions (Kis et al., 2017), and their emotional processing through mechanisms that may facilitate human–dog communication (Somppi et al., 2017).

## 4. Functional use of emotional information from human expressions

Being able to process emotional displays facilitates group cohesion (Racca et al., 2012) and allows individuals to use other's affective information to cope with events and objects in the environment (Merola et al., 2014). For instance, social referencing is the process of using social cues from another individual when facing novel stimuli and it serves as a source of approaching or avoidance information to guide one's own behaviour regarding a specific event or object (Merola et al., 2012a). A few studies have covered this aspect in domestic dogs, with evidence to support their capability of using human emotional cues via social referencing (e.g. Merola et al., 2012b), from ages as young as 8 weeks (Fugazza et al., 2018). However, while social referencing refers to the use of predictive cues to guide behaviour, it does not imply inferential processes. According to Adolphs (2002), when humans see a ‘scared’ facial expression, for example, we do not only relate it to other facial expressions in terms of its structure, but also acknowledge that the person has probably perceived something ‘scary’ and is likely to scream and run away. This information is not present in the structure of the facial expression itself; it is retrieved from past experiences with the world.

A few studies have pointed out the possibility that dogs may be able to use emotional information in a functional manner. For example, in 2013, Buttelmann and Tomasello exposed dogs to a situation where a person could behave (visually and acoustically) in a happy, disgusted or neutral manner when looking into a box and the subjects could choose one of the boxes to find hidden food. They provided important insights into the use of predictive emotional cues, as the subjects responded appropriately. In another study, Ford et al. (2019) tested dogs with the classic two-choice task, where there were two baited opaque containers that dogs must choose from with no information except the gestural cues of a person in relation to the items. In their experimental design, they presented dogs with conditions where the human directional gestural cues (looking or pointing) were conflicting, i.e. looking at one while pointing at the other. In addition, they included facial expressions of different valences (displayed by the same human signaller) to investigate which way dogs would use the social information available. They found that dogs were more likely to avoid something that had been looked at with a potentially negative face and choose the other item.

The ability to recognise emotional expressions provides individuals with the means for better adjusted interactions in various social contexts and, following Leon-Rodriguez and Sierra-Mejia (2008), the next step is recognising the possible consequences of emotional expressions. In humans, this type of knowledge usually presumes detrimental effects from negative emotions and beneficial influences of positive states. The authors suggest that being able to infer the consequences of emotions requires the establishment of causal and temporal relationships between discrete events (the emotional expressions and the potential subsequent action). Knowledge about the potential behavioural outcomes of others’ emotional experiences is crucial for making appropriate choices, reacting adequately and executing more effective social actions.

Following van Kleef et al. (2010), emotions have a great potential to shape behaviour and this applies not only to the individual level but also to social interactions and decision-making, situations when one's behaviour influences and is influenced by others’. For instance, in order to act in a strategic way, humans consider emotions as a vital source to inform their behaviour, and so will allocate their attention and efforts to it, especially when evaluating when, whether and to what extent to cooperate or to compete. In this sense, individuals use the information provided by others to guide their choices, which helps them to develop an adaptive course of action (van Kleef et al., 2010). For example, in cooperative settings, happiness might trigger approaching behaviours from the observer towards the signaller. On the other hand, according to Marsh et al. (2005), anger expressions facilitate avoidance-related behaviours, meaning that they are indeed perceived as threatening in some way. Furthermore, Farb et al. (2013) suggest an embodied cognition approach to the study of these aspects. They argue that in addition to modulating sensory inputs, emotions can reflect emotional states (cognitive and/or physiological) through observable embodied representations (musculature, posture, behaviour). The functional approach meets the embodied approach from the basis that emotional expressions are not arbitrary, i.e. particular expressions promote particular adaptive functions.

Grounded on this body of literature, Albuquerque et al. (2021) investigated dogs’ capacity to infer emotional states and emotional consequences and make functional use of indirect heterospecific emotional cues. They found that dogs are able to use the emotional information obtained from people in a functional way, i.e. they can infer the potential consequences of the displays and use that information for adjusting their own behaviour. Moreover, dogs were shown to consider the emotional information to a smaller extent when they did not need to use the human to get a desired food.

The findings presented are evidence that dogs are able to functionally use the emotional information displayed by humans when faced with a social problem. They pay attention, obtain information and use this information to adjust their behaviour. Moreover, they are able to use information previously stored in their memory from prior experiences with human emotional expressions to infer the emotional state of people.

## 5. Future directions

Other behavioural tasks could also contribute to this topic, such as discrimination tasks with the use of incongruent body-face emotional expressions. Usually, the body and face are part of an integrated system that conveys affective information and there is evidence that the presentation of a facial expression in a natural body context to human subjects allows a rapid acquisition of biologically relevant information whilst incongruent stimulus combinations may hamper categorisation (Meeren et al., 2005).

Moreover, investigating whether and how personality, attachment levels and styles, demographic factors and experience act on the motivation–emotion–cognition interface (Rosati & Hare, 2013) would provide further relevant information for the understanding of emotional communication between dogs and humans, of the affective and social lives of these domestic canids and of the evolution of social cognition as a whole.

Further research should also look into species-dependent differential responses and their underlying mechanisms. For instance, there is recent evidence showing that horses and cats are able to integrate multimodal information from facial expressions and vocalisations, i.e. they are able to recognise emotional expressions through image and sound (Nakamura et al., 2018; Quaranta et al., 2020). Thus, it is possible that other animals have the same ability and that these two species can respond to and use emotional information from human expressions in a similar way to dogs. Moreover, cultural differences and different cultural contexts must be taken into consideration if one desires to understand the dog as an entity, as a whole. In addition, looking at more than just dogs that are pets and live within a human or a multispecies family is critical. Most studies are conducted in WEIRD (Western, Educated, Industrialized, Rich, and Democratic) countries, with WEIRD dogs and people. However, other important research groups around the world are showing other facets of dogs that we did not acknowledge. This is the case for groups such as that of Bhadra (e.g. Majumder et al., 2014; Bhattacharjee et al., 2018; Brubaker et al., 2019), who have been working not only with Indian dogs, but with dogs that live on the streets, in a quite different dynamic than the usually studied subjects. This sort of aspect must be taken into account when a better understanding of the real interspecific phenomena between dogs and humans is sought.

## 6. Conclusion

There is a body of literature that explores dogs’ emotional lives, in terms of feelings, emotional expressions and emotion perception. However, the function of these abilities is far less understood. Investigating how obtaining information from others’ faces is, for instance, critical for the good adjustment of dogs in human (or multispecies) societies takes a different turn when we start to understand how this information is responded to and how this information is used. Dogs problem-solve all the time and being able to read humans’ emotional expressions, emotional states and emotionally driven behaviour is tremendously advantageous and may be seen as a highly important adaptive feature.

According to Schachter and Singer (1962), the experience of emotions can induce emotional states, which are more long lasting yet less obvious occurrences that are not time constrained to emotional expressions and could be seen as a faculty of physiological arousal and cognitive processing. As is the case for humans, the perception of emotional expressions may allow an observer to infer this subjective information about the producer of the signals (van Kleef, 2009), i.e. from the expression of the emotion, an observer can make certain decisions depending on what they have learned about that experience. Thus, in spite of the potential to expose information that can lead to vulnerability, emotions serve critical functions to organisms (Keltner & Haidt, 1999).

In addition to being able to recognise emotional expressions, dogs are able to access their affective content and respond to them. Moreover, dogs can make functional use of the emotional information they obtain from heterospecific visual emotional displays and utilise this information during decision-making. Recently, researchers worldwide have become more prone to agree that dogs’ social skills are the result of both proximal and ultimate causes and they are better understood according to an interaction prism (Resende & Garcia, 2017; Albuquerque & Savalli, 2017). Therefore, not only must a multimodal approach be taken into consideration when looking at dogs’ abilities to express and perceive emotions, but also an integrated approach must be applied to understanding how far these animals can go when they look at our emotional expressions.

## Data Availability

Not applicable.
